# Custom-Made Ion Exchange Membranes at Laboratory Scale for Reverse Electrodialysis

**DOI:** 10.3390/membranes9110145

**Published:** 2019-11-04

**Authors:** Liliana Villafaña-López, Daniel M. Reyes-Valadez, Oscar A. González-Vargas, Victor A. Suárez-Toriello, Jesús S. Jaime-Ferrer

**Affiliations:** 1 CIATEC A.C., Centro de Innovación Aplicada en Tecnologías Competitivas, Omega 201, Col. Industrial Delta, León, Guanajuato 37545, Mexico; misaelreyes812@gmail.com (D.M.R.-V.); jjaime@ciatec.mx (J.S.J.-F.); 2 Departamento de Ingeniería en Control y Automatización, Escuela Superior de Ingeniería Mecánica y Eléctrica-Zacatenco, Instituto Politécnico Nacional, UPALM, Av. Politécnico S/N, Col. Zacatenco, Alcaldía Gustavo A. Madero, Ciudad de México 07738, Mexico; ogonzalezv@ipn.mx; 3 CONACYT-CIATEC A.C., Centro de Innovación Aplicada en Tecnologías Competitivas, Omega 201, Col. Industrial Delta, León, Guanajuato 37545, Mexico; vsuarez@ciatec.mx

**Keywords:** ion exchange membranes, solvent evaporation, blue energy, reverse electrodialysis, laboratory-scale membranes

## Abstract

Salinity gradient power is a renewable, non-intermittent, and neutral carbon energy source. Reverse electrodialysis is one of the most efficient and mature techniques that can harvest this energy from natural estuaries produced by the mixture of seawater and river water. For this, the development of cheap and suitable ion-exchange membranes is crucial for a harvest profitability energy from salinity gradients. In this work, both anion-exchange membrane and cation-exchange membrane based on poly(epichlorohydrin) and polyvinyl chloride, respectively, were synthesized at a laboratory scale (255 cm^2^) by way of a solvent evaporation technique. Anion-exchange membrane was surface modified with poly(ethylenimine) and glutaraldehyde, while cellulose acetate was used for the cation exchange membrane structural modification. Modified cation-exchange membrane showed an increase in surface hydrophilicity, ion transportation and permselectivity. Structural modification on the cation-exchange membrane was evidenced by scanning electron microscopy. For the modified anion exchange membrane, a decrease in swelling degree and an increase in both the ion exchange capacity and the fixed charge density suggests an improved performance over the unmodified membrane. Finally, the results obtained in both modified membranes suggest that an enhanced performance in blue energy generation can be expected from these membranes using the reverse electrodialysis technique.

## 1. Introduction

Historically, the growing worldwide demand for energy has widely been covered by fossil fuels emitting high levels of greenhouse gases and causing accelerated global warming as a consequence. Thus, it is expected that renewables, non-intermittent, and low-carbon energy sources strengthen their position in the energy system worldwide in order to meet the world’s energy demand and progressively reverse the negative impacts caused by anthropogenic greenhouse gas emissions. Nowadays, renewables and low-carbon technologies such as wind, solar and hydropower are already incorporated into the energy matrix but its potential is limited, mainly due to unpredictable fluctuations in power production, limited efficiency and high infrastructure costs. In this context, the development of alternative power sources still necessary to replace fossil fuels as the primary energy input [1].

Salinity Gradient Power (SGP), also referred to as “Blue or Osmotic Energy,” has been identified as an attractive new energy source. The SGP concept was first proposed by Pattel [2] with the report of the “hydroelectric pile” and consists of the harnesses energy generated from the potential chemical difference when two water streams with different salt concentration are mixed [3,4,5]. SGP can be considered a renewable and immaculate energy source since only brackish water, and carbon-neutral electrical or mechanical energy is generated [3,4]. Unlike intermittent sources of solar and wind energy, the SGP can be exploited continuously and to a minor cost than that of solar or wind farms [6]. This energy source is considered abundant, since a potential between 1.4 to 2.6 TW was estimated when only considering the natural estuaries produced by the mixture of seawater and river water [7,8]. Also, energy can be generated from anthropogenic effluent, which leads to an even more significant potential for energy generation from salinity gradients [3,4,5,9,10]. However, theoretical estimates tend to decrease due to technical and operational limitations. Even so, the total global potential available to extraction is around 1 TW [3], which remains a huge amount of energy waiting to be harnessed.

There are several technologies for SGP conversion into electrical or mechanical power. However, Reverse Electrodialysis (RED) [2] and pressure retarded osmosis (PRO) [11] are the two most mature technologies, and both have been validated at pilot-plant scale [12,13]. In particular, the RED scheme consists of a set of ion-exchange membranes (IEM) stacked in an alternating pattern formed by cation- and anion-exchange membranes (CEM and AEM, respectively), with different salinity solutions filled each layer. In this scheme, the chemical potential difference causes the selective transport of ions through the CEM and AEM, from the concentrate to the diluted solution. In this case, the electro-neutrality of the solutions in each compartment is maintained via redox reaction at the anode and cathode surface, causing the transfer of electrons into an external electric circuit and electrical power can be generated. Unlike RED, PRO technology uses semipermeable membranes, which allows permeation of the solvent and retention of solute. Here, the chemical potential difference between the solutions causes transport of water from low-pressure diluted salt solution to the high-pressure concentrated salt solution. As a result, the transport of water can be used to generate electrical power. Although the theoretical potential is equal for both technologies, comparative studies between RED and PRO have concluded that RED is more effective for power generation at large-scale when seawater and river water is used as feed because of higher power density and fouling resistance [8].

In the last decade, special attention has been devoted to the study and identification of the parameters of a more significant influence on the RED process performance such as membrane development [14,15,16], process parameters [17,18,19,20], stack design [3,21,22] and fouling [23,24]. In most of these studies, it is agreed that the application of RED is mostly limited by the lack of custom membranes for RED [3,25,26]. In this sense, requirements that IEM in a RED system has to fulfill are—(1) High permselectivity (above 95% [27]), an IEM should be highly permeable to counter-ion, but not to co-ion; (2) Low electrical resistance (below 3 Ωcm${}^{2}$ [27]) an IEM should have low electric resistance so as to minimize the potential drop in the process; (3) Good mechanical and form stability, an IEM should be mechanically strong enough to construct a RED stack but thin as possible to minimize area resistance; (4) Low degree of swelling, an IEM should be low swelling when is put in contact with the ionic solutions in order to avoid the dissolution of the polymer and increase of resistances; (5) High chemical stability (at least 5 years), an IEM should be chemically stable at operating conditions; and (6) Low cost, an IEM should be as cheap as 4.3 €m^2^ to produce electricity profitably by RED technology [28].

Additionally, both electrical resistance and permselectivity of an IEM are correlated with ion exchange capacity (IEC) and fixed charge density (${\mathrm{CD}}_{\mathrm{fix}}$) [3,29,30] and are inherently related to the chemical structure of polymeric materials for IEMs [25,30]. In general, a high IEC represents high permselectivity and low electrical resistance. A high IEC value is also an indirect indicator for swelling predisposition, although this would be inherently controlled by chemical crosslinking and reinforcement of the membrane structure [3]. However, the optimal design of the IEMs represents an important challenge because there is a complex correlation between the physicochemical and electrochemical properties, which some can act to the detriment of one another. For example, the improvement of mechanical strength and permselectivity of an IEM can be achieved via the increase in the cross-linking fraction, although the increase in the electrical resistance of the membrane is a side effect. Now, if it is required, it is to reduce the electrical resistance and increase the IEC, it could be achieved by increasing the functionality of the polymer network, but it could favor the infringement to the detriment of mechanical stability and permselectivity [31,32,33]. Although there are a variety of commercial and tailor-made membranes (see compilation in Reference [3]), none of them fully meet the requirements for an efficient application in RED. Most of these were designed for fuel cells and electrolysis applications, so they show high ionic conductivity, stability, and good mechanical properties. However, these IEMs are modified by adding reinforcement additives to provide them with greater mechanical stability, causing a significant thickening of the membrane and a consequence increase in its electrical resistance [25,34,35,36]. Also, these commercial options still have a prohibitive cost for their implementation in RED on a large-scale. Additionally, the lack of available information about commercial and tailor IEM preparation, together with the variation in the test conditions, difficult its comparison and limit the conclusion about the interdependence between the IEM chemical structure and their properties. Therefore, the systematical investigation of the performance-properties-structure interdependence in IEM will allow us to design new strategies for the manufacture of IEM with suitable characteristics for RED conditions. Recently, Hong et al. [37] investigated the relationship between physical and electrochemical properties and their influence on performance in RED power generation. Here, a series of custom-made and commercial IEM were used. In this report, it was founded that a lower area resistance is a much more influential parameter on RED performance, even than permselectivity. Then, this conclusion implies that ion conductance through the IEM should be a higher priority than ion selectivity favors power generation in the RED application.

Several IEMs tailored for RED have been reported in recent years. Besides the selection of the base material, the method of preparation and modification have been key strategies for the improvement of IEM properties for RED [38]. In particular, polyepichlorohydrin (PECH) or polyvinyl chlorid (PVC) membranes are promising tailor-made membrane and surface modification is a key strategy to improve their properties. In this sense, Güler et al. [39] fabricated IEMs using PECH as active polymer backbone and a tertiary diamine (1,4-diazabicyclo[2.2.2]octane, DABCO) in dimethylsulfoxide (DMSO) as an aminating and crosslinking solution. In this case, PECH membranes were blended with poly(acrylonitrile) (PAN) at PECH/PAN ratios between 0.1 to 1.0. Above PECH/PAN = 0.3, a significant increment in area resistance was evidenced and correlated with an increase in the density of available ion-exchanges groups. In turn, the decrease in permselectivity was associated with a higher swelling degree. Other works have reported notable improvement of selectivity in IEM by grafting of polyelectrolytes on the material surface [40]. For AEM, increases in selectivity have been achieved by the modulation of surface hydrophilicity through the covalent bonding layer-by-layer assembly [41]. On the other hand, heterogeneous IEM are cheaper than homogeneous ones, hence they can be used as a base for the development of even cheaper materials with enhanced physical and electrochemical properties for RED. In this sense, PVC is a flexible polymer with a good thermal, biological, and chemical resistance. Also, its low price makes it more attractive to be used as a raw material in membrane manufacturing. Previously, Hosseini et al. [42] used a blend of PVC and cellulose acetate (CA) as a material base to prepare a heterogeneous CEM for an electrodialysis application. In this case, the decrease of the PVC/CA ratio was related to a significant enhance of IEC. In this case, PVC membranes can be considered as a good option for RED application but electrochemical properties need to be improved.

In this work, the effect of membrane modification and up-scaling from bench to lab-scale were investigated. For this, reported homogeneous PECH anion-exchange membrane [39] and heterogeneous PVC cation-exchange membrane [43] were synthesized as used as a reference. In order to improve the performance of the synthesized CEM, PVC with medium molecular weight was selected. For AEM modification, the glutaraldehyde-induced (PEI) multilayer deposition was used.

## 2. Materials and Methods

For the preparation of the AEM, polyepichlorohydrin (PECH; ${\left(CH\left(CH_{2}Cl\right)CH_{2}O\right)}_{n}$; average molecular weight, $M_{w}$ ∼ 700,000), polyacrylonitrile (PAN; ${\left(C_{3}H_{3}N\right)}_{n}$; $M_{w}$ ∼ 150,000), 1,4-Diazabicyclo[2.2.2]octane (DABCO® 33-LV; $C_{6}H_{12}N_{12}$; $M_{w}$ ∼ 112.17 g mol^−1^ and density, ρ = 1.02 g mL^−1^) and dimethyl sulfoxide (DMS; ${\left(CH_{3}\right)}_{2}SO$≥ 99.5%; $M_{w}$ 78.13 g mol^−1^; ρ = 1.10 g mL^−1^) were used. Polyethylenimine (PEI; $(C_{2}H_{5}N)n$; $M_{w}$ ∼ 25,000) and glutaraldehyde solution (GA; $C_{5}H_{8}O_{2}$; Grade I 25 in H${}_{2}$O) were used for the AEM modification. All these reagents were acquired from Sigma Aldrich.

For the preparation of the CEM, polyvinyl chloride (PVC; ${\left(C_{2}H_{3}Cl\right)}_{n}$; $M_{w}$∼ 43,000; ρ=1.4g mL^−1^, and inherent viscosity, μ=0.51g mL^−1^); tetrahydrofuran anhydrous (THF $C_{4}H_{8}O\geq 99.9\%$, with a $M_{w}$ of 72.11 g mol^−1^ and density of 0.89 dLg^−1^); and a strongly acidic cation exchange resin (Amberlyst® 15 hydrogen form, dry) all these reagents were acquired from Sigma Aldrich. The cellulose acetate (CA, $C_{164}H_{174}O_{111}$; $M_{w}$∼ 100,000; ACROS Organics) was used for the modification of this membrane.

### 2.1. Preparation of Ion Exchange Membranes

#### 2.1.1. Anion Exchange Membrane

The AEM was prepared using the casting solution technique described in Reference [39]. We prepared solutions of PECH (20%), PAN (12%) and DABCO (12.25%) in DMSO, which were kept in constant agitation at room temperature until complete dissolution. PECH:PAN:DABCO (2:1:2) were mixed in a three-neck round-bottom flask under constant stirring at 80 ${}^{\circ }C$ for 30 min using a unjacketed distilling column. This mixture was cast onto a glass plate and placed in a mechanical convection oven inside a hermetically sealed glass box at 110 ${}^{\circ }C$ for 2 h. Afterwards, the seal was removed and the solvent was allowed to evaporate at 130 ${}^{\circ }C$ for another 30 min. The glass plate with the membrane was placed in the extraction hood for cooling, after which it was immersed in a NaCl 0.5 M solution for a few minutes in order to detach the membrane. The membrane was stored submerged in the same NaCl solution.

This AEM was also modified [41] using PEI and GA as superficial modifier agents in order to increase the anionic selective separation, surface homogeneity, thermal and mechanical stability and reduce the membrane swelling [41,44,45]. The superficial modification of the AEM was performed once the previously described AEM was cooled and detached from the glass plate, it was then alternately immersed (3 times) in PEI (2 g L^−1^ NaCl) and GA (10%) for 30 min each time. The immersion process allows for a total surface modification on both sides of the AEM. This modified membrane was designated modified-AEM and was stored in a NaCl solution.

#### 2.1.2. Cation Exchange Membrane

We prepared the CEM using the casting solution technique described in reference [43]. Prior to synthesis, the cation exchange resin was mechanically crushed and dried at 30 ${}^{\circ }C$ for 48 h. This crushed resin was sieved using a mesh, and the particles between 38 μm to 45 μm were collected. For the membrane preparation, we dissolved the PVC in the THF (1:20, *w*/*v*) using a glass reactor and stirred for 4 h. We then stirred in a stoichiometric amount of the previously collected cation exchange resin (1:1 *w*/*w*), followed by an ultrasonic bath for 30 min. Lastly, the solution was stirred for another 15 min. The whole preparation of the polymeric mixture was carried out at room temperature. We cast this mixture onto a glass plate, leaving it to dry at room temperature until total solvent evaporation. We submerged the membrane into NaCl 0.1 M solution to facilitate removal from the glass plate; the membrane was stored in this same solution.

We then carried out the membrane modification [46] using CA as modifier agent mainly because of its high hydrophilicity, good biocompatibility and toughness, high potential flux, solvent resistance, and low cost [46,47]. The CA was directly added to the polymer solution during the synthesis process allowing a complete structural modification of the membrane. This new membrane was designated modified-CEM, and it was prepared by mixing PVC and CA 1:2 (*w*/*w*) in THF. We added the crushed cation exchange resin (38 μm to 45 μm) and then followed the same preparation procedure described above for the CEM. This modified membrane was also stored in a NaCl solution.

### 2.2. Membrane Characterization

#### 2.2.1. Fourier-Transform Infrared Spectroscopy (FTIR)

Surface characterization is essential to verify both the structure of the synthesized membrane and the crosslinking between reagents. Fourier-transform infrared (FTIR) spectra in transmittance mode were collected in a Thermo Scientific™ Nicolet™ iS10 FTIR spectrometer using the OMNIC software suite, which allowed us to collect and optimize the measured spectra. The FTIR spectra were measured in five different sections on both sides of the membrane in order to verify the presence of the different chemical groups.

#### 2.2.2. Scanning Electron Microscopy (SEM)

The morphology of the membranes was determined through scanning electron microscopy (SEM) images obtained using both a JSM-7800F Schottky and a Carl Zeiss SIGMA-HDVP field emission scanning electron microscopes. Before sample analysis, the membranes were placed on the sample holders and were coated with gold, thus allowing the membrane surface observation.

#### 2.2.3. Ultrapure Water Contact Angle

The ultrapure water contact angle ($\theta_{w}$) quantifies the wettability of a solid surface. The ultrapure water used has a conductivity of 0.203 μS cm^−1^ ± 0.089 μS cm^−1^ and a pH of 8.35 ± 0.49 at 25 ${}^{\circ }C$. $\theta_{w}$ on both sides of all the synthesized membranes were obtained using the DataPhysics OCA 15EC in sessile drop mode. Before the $\theta_{w}$ measurement, the membrane was dried in a convection oven for 48 h at 35 ${}^{\circ }C$, followed by 24 h in a desiccator. The dried membranes were fastened the membranes on glass slides using double-sided tape. The ultrapure water drops were dosed using a Hamilton glass syringe with a maximum volume of 500 μL, along with a stainless steel needle (outside diameter of 0.53 mm ± 0.01 mm). The approximate volume of each drop was 3.25 μL ± 0.18 μL. $\theta_{w}$ was measured 166 ms after drop deposition. Finally, the left and right contact angles of each drop were calculated from the digitized image using an elliptical adjustment. 10 droplets of ultrapure water were deposited on different sections on both sides of the same membrane sample, with an experimental error value of ±3${}^{\circ }$ [48].

#### 2.2.4. Swelling Degree

The swelling degree (SD) is a parameter that depends on the ion exchange groups in contact with solvents, especially water. The SD can be calculated considering the mass change between the dry and the hydrated membrane; these masses are related through the following equation [39],

(1) $$\mathrm{SD}\;\left(\%\right)=\frac{w_{\mathrm{wet}}-w_{\mathrm{dry}}}{w_{\mathrm{dry}}},$$

where $w_{\mathrm{wet}}$ is the weight of the hydrated membrane and $w_{\mathrm{dry}}$ is the weight of the dry membrane, both in grams. In order to completely dry the membrane, we inserted it into the mechanical convection oven at 45 ${}^{\circ }C$ for 12 h, followed by 24 h in the desiccator. After that, we weighed the membrane on the Sartorius™ Cubis™ analytical balance (Series MSA124S), which has a resolution of 0.1 mg, a linearity of ≤±0.2 mg and a repeatability of ≤±0.1 mg. The same membrane was hydrated with deionized water for 12 h, removing the excess water with filter paper and weighing it on the same analytical balance.

#### 2.2.5. Ion Exchange Capacity

The ion-transport effectiveness of the IEM can be measured through the ion exchange capacity (IEC), which is the number of fixed charges inside the IEM per unit weight of dry polymer [49,50]. To measure the IEC of the synthesized membranes we used the Fisher and Kunin method [51]. The membranes were dried for 24 h at 35 ${}^{\circ }C$, then placed overnight in a desiccator. The samples were weighed using an analytical balance and were cut into the smallest possible pieces. Subsequently, the CEM and AEM were immersed in 1 M HCl and 1 M NaOH solutions, respectively, and left untouched overnight in order to achieve equilibrium conditions. The membranes were rinsed to remove excess solution and were finally titrated using a volumetric solution of 0.01 M HCl or 0.01 M NaOH using the 916 Ti-Touch (Metrohm) and a pH electrode (Metrohm 6.0280.300) for specific use with aqueous acid-base titration.

#### 2.2.6. Charge Density

In a reverse electrodialysis system, the electrical resistance and the permselectivity of the membrane are essential properties, since they are directly related to the maximum obtainable power; in turn, these properties can be determined through the density of fixed charges (${\mathrm{CD}}_{\mathrm{fix}}$) of the membrane [50]. ${\mathrm{CD}}_{\mathrm{fix}}$ represents the milliequivalents of fixed groups by volume of water in the membrane, and can be calculated from the SD and IEC as

(2) $${\mathrm{CD}}_{\mathrm{fix}}=\frac{\mathrm{IEC}}{\mathrm{SD}}.$$

#### 2.2.7. Permselectivity

Membrane permselectivity (S) describes the degree to which ion exchange membranes exclude co-ions. A perfect membrane has no co-ion transport and hence a permselectivity of one, while a membrane with no ionic selectivity transports co-ions at the same rate as the solution and has a permselectivity of zero. The permselectivity was determinated via membrane potential approach. The membrane potential was measured using a Scribner H-cell (1/2 cell volume = 30 mL) which is a two compartment cell with Luggin probes where a membrane sample is placed between two solutions with two different concentrations. A high concentration (0.5 M) and a low concentration (0.1 M) solution is pumped on either side of the membrane. Two reference electrodes (Ag/AgCl gel) were used to measure the potential over the membrane. The system is allowed to reach equilibrium at 25 ${}^{\circ }C$. Once equilibrium is obtained, the potential across the reference electrodes is averaged over the span of 15 min to yield the measured membrane potential. The experimental potential was monitored by using a BioLogic VSP-300 potentiostat.

The permselectivity was calculated from the ratio of the experimental potential over the theoretical potential. The theoretical potential difference in the system was calculated with the Nernst equation. The permselectivity (%) was determinated using the equation:

(3) $$S\;\left(\%\right)=\frac{\Delta E_{\exp}}{\Delta E_{\mathrm{th}}},$$

where $\Delta E_{\exp}$ is the measured membrane potential (mV) and $\Delta E_{\mathrm{th}}$ is the theoretical membrane potential (mV).

#### 2.2.8. Electrical Resistance

The electrical resistance (ER) of the membrane was measured by electrochemical impedance spectroscopy (IES) using a four-electrode H-Cell configuration. The four-probe configuration is the most convenient if the membrane is in contact with a liquid electrolyte because it can eliminate the contributions from the electrode injecting stimulus and the electrolyte charge transfer resistance from the impedance spectra, thus allowing complete focus on the membrane and interfaces [52]. Impedance spectra were obtained over a frequency range of 1 MHz to 50 Hz using a BioLogic VSP-300 potentiostat.

For the measurements, a membrane is placed in an H-cell with an electrolyte solution of NaCl 0.5 M. At high frequencies the intercept on the real axis gives the membrane resistance ($R_{m}$) or the membrane plus solution resistance ($R_{m+s}$). In practice, the solution resistance can be obtained with blank experiments (without the membrane) and its contribution can be subtracted to obtain the pure membrane resistance. The ER was determined using the equation:

(4) $$\mathrm{ER}\;\left(\Omega \;{\mathrm{cm}}^{2}\right)=\left(R_{m+s}-R_{m}\right)\times A$$

where *A* is the membrane surface area (cm).

## 3. Results and Discussions

### 3.1. Membranes Characterization

**Figure 1:**
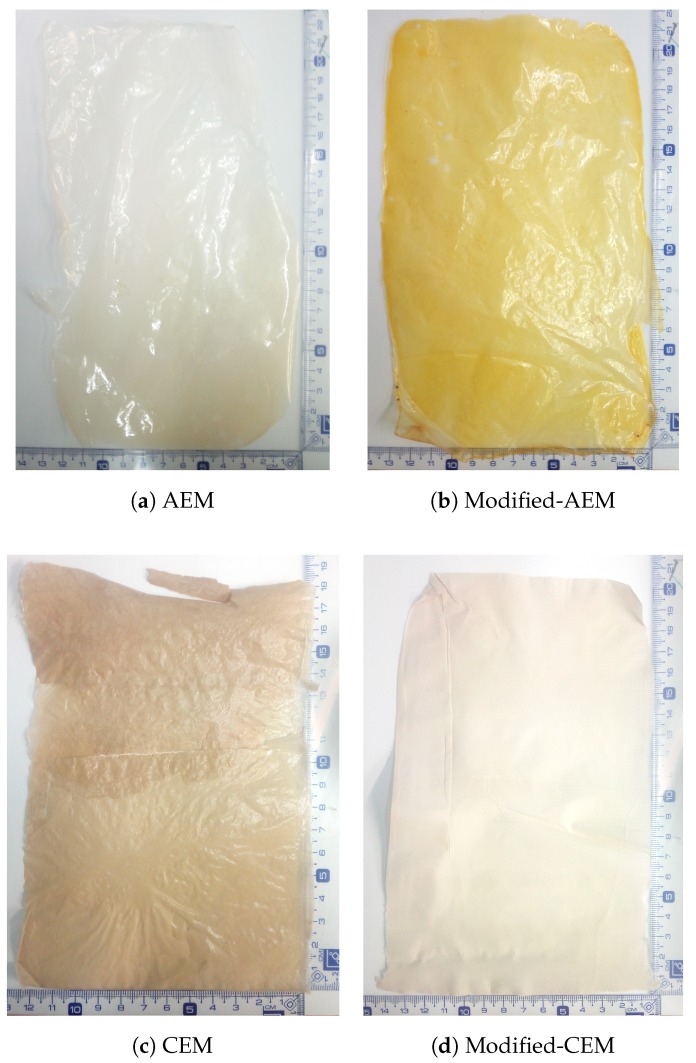
Ion exchange membranes synthesized by solvent evaporation with and without modification.

Preliminary work consisted of preparing four AEMs and four CEMs at bench scale, comprised of materials based on reported results, accessibility, and cost-effectiveness. The membranes with the best performance and reproducibility were selected to be upscaled and these were the homogeneous AEM and the heterogeneous CEM used throughout this work. We verified the chemical membrane resistance of the synthesized membranes through the pH variation. This pH resistance test was evaluated by submerging a membrane in ultrapure water adjusted to pH values between 3 and 13 for 24 h. After this time, all membranes maintained their mechanical integrity and physical characteristics, suggesting a successful and apparent crosslinking between the polymeric base binder and the active polymer. In Figure 1, we present photos of both IEMs before and after modification. All the membranes were synthesized with dimensions around 255 cm^2^. Note the difference in the membrane color after the modification, which indicates changes in the membrane characteristics.

#### 3.1.1. FTIR Spectroscopy

**Figure 2:**
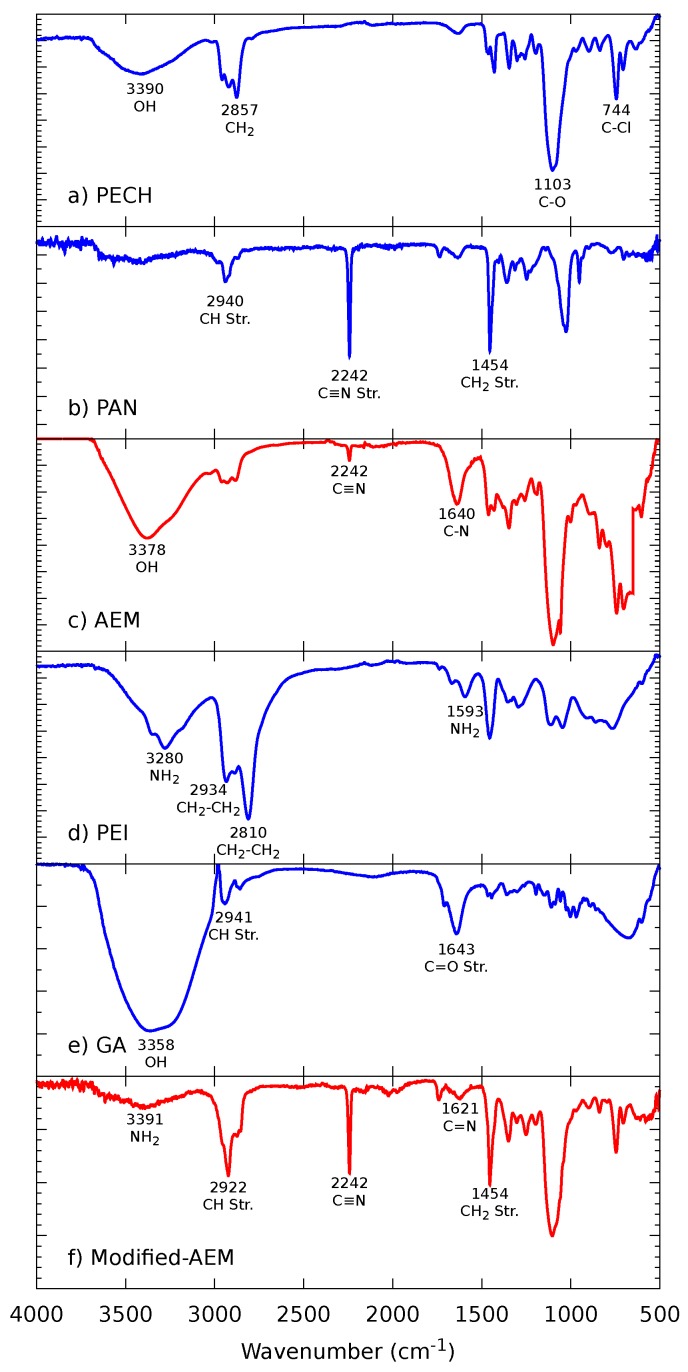
Fourier transform infrared (FTIR) spectra of the (**a**) polyepichlorohydrin (PECH) and (**b**) poly(acrylonitrile) (PAN) reagents, and the (**c**) synthesized anion exchange membrane (AEM), followed by the modified-AEM synthesized by solvent evaporation.

We used FTIR spectroscopy to confirm the crosslinking reaction between the different reagents used in the synthesized membranes. No difference was observed between the different FTIR spectra obtained from either side of each IEM. In Figure 2 we present the FTIR spectrum of the different reagents used to synthesize the AEMs before and after modification. First, the spectrum of PECH and PAN are presented with their principal peaks [53,54] (panels a and b), followed by the synthesized AEM in panel c. The three characteristic peaks verify the proper crosslinking of the polymers above the first peak, derived from the PECH, corresponds to the OH group at 3378 cm^−1^; the second peak, at 2242 cm^−1^ belongs to the C-N of the PAN polymer; lastly, the third peak at 1640 cm^−1^ is associated with the C-N group that also corresponds to the PECH polymer [39]. This membrane was superficially modified immersing it alternately in PEI and GA solutions. The spectra of both modifying solutions are shown after the AEM (panels d and e), along with their principal peaks [55,56,57,58]; finally, panel f presents the spectrum for the modified-AEM. The GA can react with the primary and secondary amines of the PEI; this process allows the assembling of the subsequent PEI layers [41,59]. For this reason, the peak at 3391 cm^−1^ appears reduced in the modified-AEM spectrum. The crosslinking with the GA also makes the peak at 2922 cm^−1^ more intense [60]. We can also observe two peaks that belong to the PAN polymer; the first at 2242 cm^−1^, which is part of the AEM and remains at the same wavenumber, and the second at 1454 cm^−1^ which belong to CH${}_{2}$ stretch group. The same reaction between the GA and the amines of PEI provoke a weak peak at 1621 cm^−1^, indicating the formation of the C=N group and reagent crosslinking [44,60,61].

**Figure 3:**
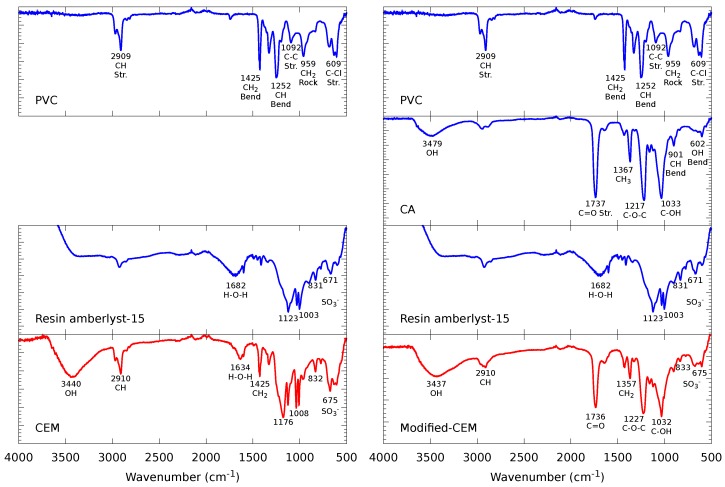
FTIR spectra of polyvinyl chlorid (PVC), cellulose acetate (CA) and pure resin Amberlyst-15 reagents, with the synthesized cation exchange membrane (CEM) and modified-CEM synthesized by solvent evaporation.

Figure 3 presents the FTIR spectrum of the CEMs divided into two columns with the different reagents used to synthesize the unmodified (left column) and structurally modified (right column) membrane. The first column features the different reagents that form the CEM, starting with the PVC and resin Amberlyst-15 spectra with their principal peaks [62,63,64]. For the final synthesized membrane, there is a peak at 3440 cm^−1^ corresponding to the OH group; two peaks at 2910 cm^−1^ and 1425 cm^−1^ are derived from the PVC polymer and were detected at the same wavenumber but with less intensity. Finally, the peaks at 1634 cm^−1^, 1176 cm^−1^, 1008 cm^−1^, 832 cm^−1^, and 675 cm^−1^ correspond to the resin Amberlyst-15; however, these peaks appear to be shifted to slightly higher wavenumbers, suggesting an adequate crosslinking between the PVC and the resin Amberlyst-15 [43,65].

The second column also shows the PVC and resin Amberlyst-15 spectra along with the spectrum for CA, the structural modifier agent, with its principal peaks [63,66]. The modified-CEM presents a peak at 3437 cm^−1^ that corresponds to the OH group; there is also a peak at 2910 cm^−1^ corresponding to the CH stretch of the PVC. The peaks observed at 1357 cm^−1^ and 1227 cm^−1^ belong to the CA and are slightly shifted in wavenumber, along with two other peaks at 1736 cm^−1^ and 1032 cm^−1^ with no change in their characteristics. Finally, SO${}_{3}^{-}$ peaks from the resin Amberlyst-15 are present at lower wavenumbers. The observed peaks, and the absence of the peak at 1425 cm^−1^ of the PVC, suggest crosslinking between the different reagents.

#### 3.1.2. Scanning Electron Microscopy (SEM)

**Figure 4:**
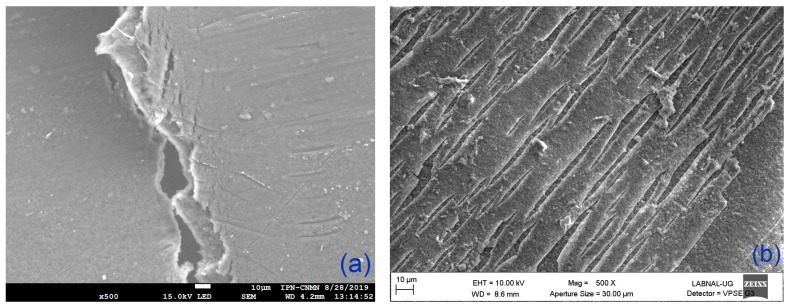
Scanning electron microscopy (SEM) images of the superficial view of (**a**) AEM (500×) and (**b**) Modified-AEM (500×).

We used SEM images to compare and verify the structural and superficial modification of the IEMs. Because the FTIR and contact angles results showed similar properties for either side of the membranes, the images were were taken randomly from only one side of the membrane. Figure 4 shows the SEM images of the AEM and modified-AEM. The AEM presents a very homogeneous surface without pores; the membrane fracture observed in this image shows a homogeneously dense matrix. After the superficial modification with PEI and GA, we observe a uniformly cracked layer on the membrane surface.

**Figure 5:**
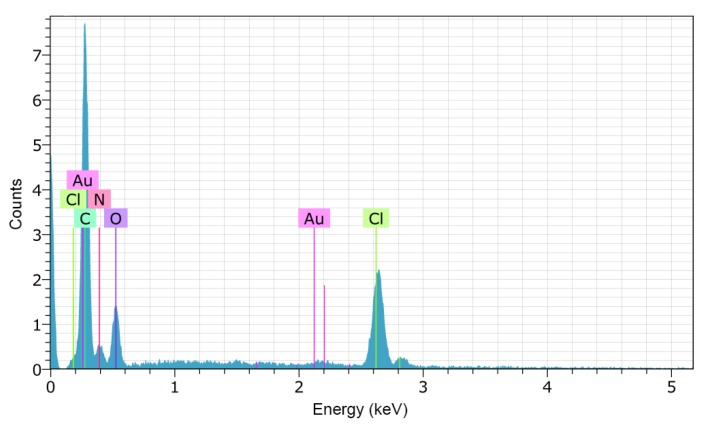
Energy Dispersive X-ray Spectroscopy (EDS) analysis results for the modified-AEM.

The chemical microanalysis of the AEM and the modified-AEM indicates the presence of the same elements, which is expected because the reagents for the modification share the same elements as the reagents used in the base membrane. In Figure 5 we present the chemical microanalysis of the modified-AEM obtained from Energy Dispersive X-ray Spectroscopy (EDS); the presence of nitrogen from PAN and DABCO, and oxygen and chlorine from PECH can be observed.

**Figure 6:**
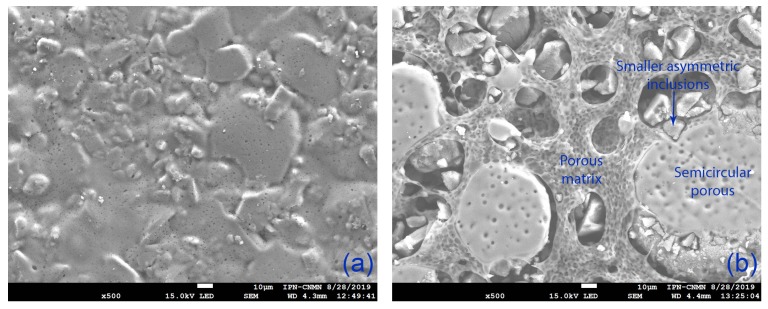
SEM images of the superficial view of (**a**) CEM (500×) and (**b**) Modified-CEM (500×).

Figure 6 shows the SEM images for the CEM and modified-CEM. We observe a heterogeneous and uniformly distributed surface in the CEM; the modified-CEM has a rougher surface with distinct structures, comprised namely of a porous matrix with large semicircular porous surfaces, and smaller asymmetric inclusions.

**Figure 7:**
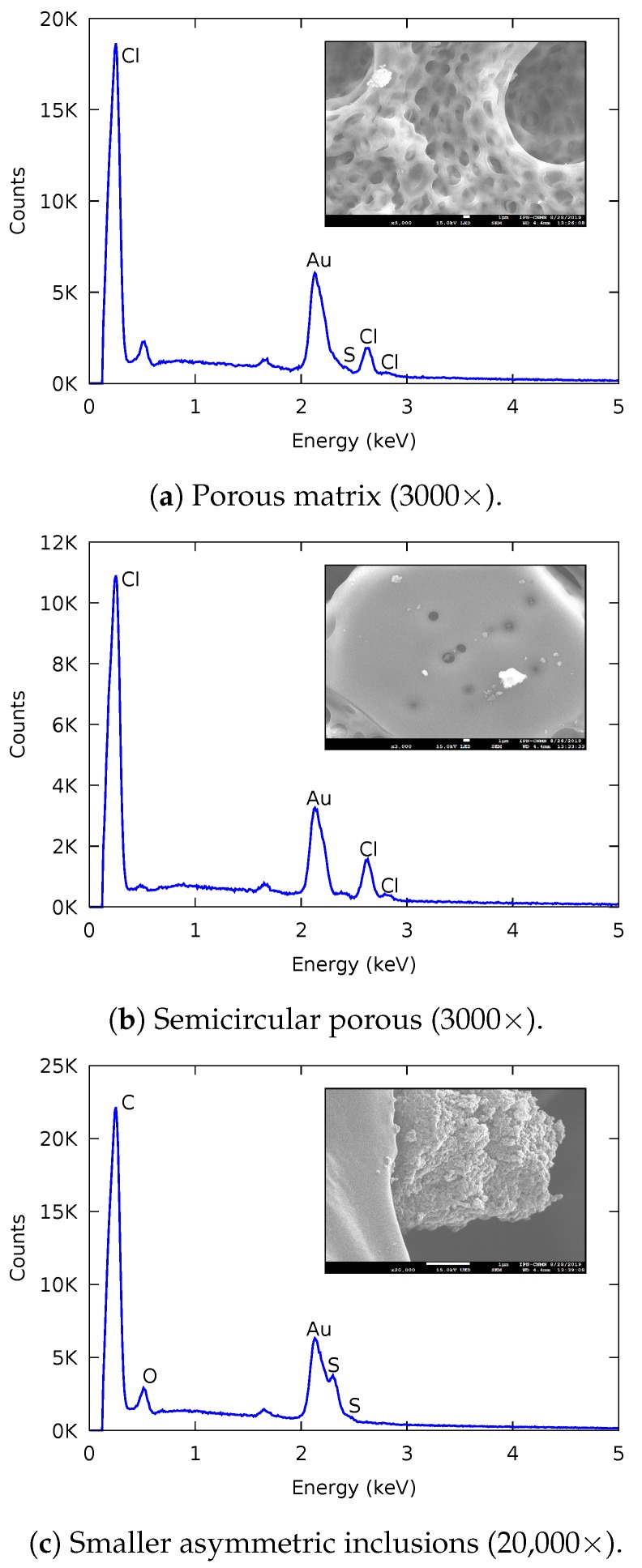
EDS analysis results for the modified-CEM.

Figure 7 shows the different structures of the modified-CEM along with their chemical microanalysis from EDS. The changes in this membrane are due to the addition of cellulose acetate which generates a significant structural modification. We can identify the characteristic elements of each structure; any gold present is from the sample preparation for the SEM. In the porous matrix, sulfur and chlorine are present from the resin Amberlyst-15 and PVC. A high percentage of chlorine in the large semicircular porous surfaces suggests the presence of PVC. Finally, the smaller asymmetric inclusions inside the porous matrix contain sulfur and oxygen, corresponding to the resin Amberlyst-15 and the CA. These changes suggest an adequate insertion of the CA through the structural modification of the CEM.

#### 3.1.3. Ultrapure Water Contact Angle and Membrane Thickness

The wettability of the membrane surface plays an important part in determining the membrane performance characteristics. This parameter is directly related to the permeated flux, the rejected flux, and the membrane fouling; and can be determined from contact angle ($\theta_{w}$) measurements. The images used for the $\theta_{w}$ calculation were also used to measure the membrane thickness; these values are reported in Table 1 and represent the average value (and standard error) of ten independent measurements on both sides of the different synthesized membranes using the same chemical composition.

Materials are highly hydrophilic when $\theta_{w}<45{}^{\circ }$, hydrophilic/hydrophobic when $45{}^{\circ }<\theta_{w}<90{}^{\circ }$, and hydrophobic when $\theta_{w}>90{}^{\circ }$ [67]. The AEM shows hydrophilic/hydrophobic properties before modification with an increase in hydrophilicity after the addition of the modifier agents GA/PEI, due to deposition of a PEI layer on the membrane surface without significantly impacting the surface roughness [41]. The thickness of both modified membranes increased, which is related to the modification process. It is important to control the thickness of the synthesized membranes as lower membrane thickness enhances ion transport [68]. Commercial and custom-made IEMs report values ranging from 33 μm to 764 μm [68]. Our membranes are in the lower part of this range; however, membrane thickness has no significant impact on the IEC, the SD, and the permselectivity when the membranes are comprised of the same reagents [68]. Therefore, we can measure these parameters for our membranes disregarding any adverse effects from the increased thickness.

Previously reported contact angles for the CEM, between 103${}^{\circ }$ to 110${}^{\circ }$ [46,65,69], indicate a hydrophobic surface. Our synthesized membrane was consistent with the reported behavior, with value of $\theta_{w}=99\pm 3^{\circ }$. After the addition of CA, the contact angle was expected to decrease due to the high hydrophilicity of this polymer [46]. However, the hydrophobicity increased ($\theta_{w}=134\pm 1^{\circ }$), which is probably related to the surface roughness [70] of the membranes obtained with the structural modification, which was observed in the SEM images. The hydrophobicity could probably be improved by varying the relationship between the PVC and the CA.

#### 3.1.4. Swelling Degree, Ion Exchange Capacity, Fixed Charge Density, Permselectivity, and Electrical Resistance

Table 2 presents the swelling degree (SD), ion exchange capacity (IEC), fixed charge density (${\mathrm{CD}}_{\mathrm{fix}}$), permselectivity (S), and electrical resistance (ER) of the synthesized AEM before and after modification. We also include reference values found in the literature, and values for two commercially available AEMs.

Our synthesized AEM (surface area of 255 cm^2^) and the reference AEM [39] are very similar, suggesting that the membrane performance is comparable despite the different membrane size. The SD of the synthesized AEM is smaller than the reported value, suggesting a reduced water uptake, while the IEC presents a very similar amount of ion exchange groups per dried membrane. ${\mathrm{CD}}_{\mathrm{fix}}$ is slightly higher, indicating a slight increase in the permselectivity. After the membrane modification with GA/PEI, there is a decrease in the SD and an increase of both the IEC and the ${\mathrm{CD}}_{\mathrm{fix}}$, indicating that the modified-AEM is capable of delivering better performance over the unmodified membrane.

The increased IEC is directly related to the increased membrane hydrophilicity and ionic salvation; however, this can also lead to high values of SD and conductivity along with mechanical weakness [72]. Nevertheless, we observed a decrease in the SD suggesting a limited introduction of water molecules in the membrane matrix, despite presenting a more hydrophilic surface. We can attribute this phenomenon to the GA that provides hydrophobic crosslinking points between the layers of the superficial modification. Finally, the increase in ${\mathrm{CD}}_{\mathrm{fix}}$ indicates an increase in the membrane permselectivity. This increase can be explained by the adjustment in the hydrophobicity-hydrophilicity relation in the membrane [41]. Finally, with the PEI modification of the membrane the properties are similar to those of the Fujifilm Type 10 AEM [71].

Table 3 presents the SD, IEC, ${\mathrm{CD}}_{\mathrm{fix}}$, *S*, and ER of the synthesized CEM before and after modification. We also include reference values found in the literature, and values for two commercially available CEMs.

Comparing both synthesized CEMs and our measured IEC is comparable with the reported value, which indicates a similar amount of ion exchange groups per dried membrane; however, our SD value is significantly smaller. In general, high water content can provide more and wider transfer channels for the co- and counter-ion transportation, and decreases ion selectivity. These performance differences could be caused by the reagent characteristics, specifically the PVC used as a polymeric based binder. The molar mass of the PVC can have a significant influence on the structure and transport rate characteristics of PVC membranes. In ultrafiltration membranes, increasing molar mass can decrease porosity, mean radius, and pore volume [73]. We can assume that the difference between our membranes and membranes reported in the literature (often with unknown molar mass) may be entirely due to this fact. Comparing both synthesized CEMs, we can observe that the three parameters reported in Table 3 are slightly higher for the modified-CEM than in the CEM. These differences are due to the hydrophilic characteristics of the CA, even if the overall membrane has a higher hydrophobicity. The hydrophilicity of CA generates a higher adsorption of water molecules, increasing the SD with a possible loss of mechanical strength; such water adsorption facilitates ion transportation which increases the IEC value, and thus the permselectivity ${\mathrm{CD}}_{\mathrm{fix}}$.

## 4. Conclusions

In this work, poly(epichlorohydrin) based anion exchange membrane and poly(vinylchloride) based cation exchange membrane were prepared at laboratory scale (255 cm^2^) via solvent evaporation technique. Superficial and structural modifications were performed on the poly(epichlorohydrin) based anion exchange membrane and the poly(vinylchloride) based cation exchange membrane, respectively. For the anion exchange membrane modification, poly(ethylenimine) and glutaraldehyde were used as modifier agents. Cellulose acetate was used to performed the cation exchange membrane modification. The superficial modification of the anion exchange membrane with poly(ethylenimine) allows a decrease of the water content in the membrane, which in turn increases the fixed-charge density and permselectivity. The structural modification on the cation exchange membrane was verified through scanning electron microscope images along with their elemental analysis. The characterization performed in the modified cation exchange membrane suggests a more hydrophobic surface along with a slight increase in membrane water content, ion transportation, and permselectivity in comparison with the unmodified cation exchange membrane. The results obtained in both modified membranes suggest that an enhanced performance for blue energy generation can be expected from these membranes using the reverse electrodialysis technique.

## Figures and Tables

**Table 1:** Ultrapure water contact angle ($\theta_{w}$) and membrane thickness values for the ion exchange membranes (IEMs) synthesized in this work (SE n=10).

Membrane	$\theta_{w}$ ± SE	Thickness ± SE
	(${}^{\circ }$)	(μm)
AEM	81 ± 2	77 ± 3
Modified-AEM	58 ± 1	154 ± 1
CEM	99 ± 3	109 ± 2
Modified-CEM	134 ± 1	269 ± 2

**Table 2:** Swelling degree (SD), Ion Exchange Capacity (IEC), Fixed charge density (${\mathrm{CD}}_{\mathrm{fix}}$), Permselectivity (*S*), and Electrical resistance (ER) with standard error (SE, *n* = 3) of the synthesized AEM before and after modification. We also include reference values found in the literature [39], and values for two commercially available AEMs, an AMX and a Fujifilm Type 10 AEM [3,71].

Membrane	SD ± SE	IEC ± SE	${\mathrm{CD}}_{\mathrm{fix}}$ ± SE	*S*± SE	ER ± SE
	(%)	(meq g^−1^)	(meq g^−1^)	(%)	(Ω cm${}^{2}$)
AEM *	30.1 ± 1.1	1.4 ± 0.1	4.5 ± 0.4	87.0 ± 0.4	1.3 ± 0.3
Modified-AEM *	25.0 ± 2.1	2.5 ± 0.3	10.1 ± 1.2	94.9 ± 0.2	0.9 ± 0.1
AEM ^†^	49.1 ± 0.21	1.68 ± 0.04	3.42 ± 0.06	87.0 ± 0.01	1.32 ± 0.16
AMX ^‡^	16	1.25	5.4	90.7	2.35
Fujifilm Type 10 AEM ^‡^	23	1.5	6.52	94.5	1.5

* This work. ^†^ Reference. ^‡^ Commercial.

**Table 3:** Swelling degree (SD), Ion Exchange Capacity (IEC), Fixed charge density (${\mathrm{CD}}_{\mathrm{fix}}$), Permselectivity (*S*), and Electrical resistance (ER) with standard error (SE, n=3) of the synthesized CEM before and after modification. We also include reference values found in the literature [43,46], and values for two commercially available CEMs, an CMX and a Fujifilm Type 10 CEM [3,71].

Membrane	SD ± SE	IEC ± SE	${\mathrm{CD}}_{\mathrm{fix}}$ ± SE	*S*± SE	ER ± SE
	(%)	(meq g^−1^)	(meq g^−1^)	(%)	(Ω cm${}^{2}$)
CEM *	4.0 ± 0.1	1.3 ± 0.2	33.6 ± 4.8	90.0 ± 0.3	6.77 ± 0.14
Modified-CEM *	4.5 ± 0.7	1.6 ± 0.1	36.6 ± 3.0	90.9 ± 0.7	3.10 ± 0.32
CEM ^†^	25	1.45	5.8	79	23
Modified-CEM ^†^	43	1.53	3.5	67	12
CMX ^‡^	18	1.62	8.8	99	2.91
Fujifilm Type 10 CEM ^‡^	21	1.67	7.95	94.7	2.3

* This work. ^†^ Reference. ^‡^ Commercial.
